# Stability evaluation of [^18^F]FDG: literature study, stability studies from two different PET centres and future recommendations

**DOI:** 10.1186/s41181-022-00154-3

**Published:** 2022-02-24

**Authors:** Jes G. Holler, Børge Renmælmo, Richard Fjellaksel

**Affiliations:** 1 Department of Nuclear Medicine, The PET Imaging Centre, University Hospital of Central Norway, Ragnhilds gate 15, 7030 Trondheim, Norway; 2 The PET Imaging Centre, University Hospital of North Norway, Hansine Hansens veg 82, 9019 Tromsø, Norway; 3 Department of Health and Care Sciences, UiT The Arctic University of Norway, Hansine Hansens veg 18, 9019 Tromsø, Norway

**Keywords:** [^18^F]FDG, Stability, Quality control, Physiochemical aspects, Drug product development, Radiopharmaceutics, Pharmaceutics, Manufacturing process design

## Abstract

**Background:**

The need for a stability evaluation of [^18^F]FDG is evident. The main purpose of this study was to make recommendations for determining the shelf life based on the available stability literature and our own two-centre stability studies.

**Results:**

We performed a non-systematic literature study to find the most relevant stability data for [^18^F]FDG. The amount of radioactivity, radio-stabilizers, choice of synthesis, dilution, pH, temperature, storage and the choice of stability tests and acceptance criteria were the most important factors to evaluate for the implementation of good manufacturing practice. Moreover, we discuss some limitations of the study, especially the choice of synthesis, photostability, the environment, temperature and storage. Based on these data, we designed our own two-centre stability studies. All the defined acceptance criteria were met.

**Conclusions:**

We have made recommendations for future stability evaluations based on our findings. The most important findings were that the amount of the radio-stabilizer ethanol should be > 0.1 % ethanol for activities up to 4 GBq/mL and > 0.2 % ethanol for activities up to 22.7 GBq/mL to keep [^18^F]FDG stable.

## Background

There is a need for an [^18^F]FDG stability evaluation guide/checklist for radiopharmaceutical production sites. [^18^F]FDG has been adopted worldwide as the most widely used clinical positron emission tomography (PET) radiotracer since the first in human PET imaging in 1976 (Hess et al. 1976). There are several stability studies available. Hung (2002) compares different quality tests and demands in the United States Pharmacopeia) USP/European Pharmacopeia (Ph. Eur.)/The draft Chemistry, Manufacturing, and Controls (CMC) issued by the U.S. Food and Drug Administration (FDA) and Yu (2006) uses BP (British Pharmacopeia) as a quality reference standard (Hung 2002; Yu 2006). However, there is no review on updated stability evaluation based on available literature, recommendations and implementation for good manufacturing practice (GMP). Herein, we present a non-systematic literature study, stability studies of [^18^F]FDG from two different PET centres and our recommendations for future [^18^F]FDG implementations for radiopharmaceutical production sites.

The stability of [^18^F]FDG is of high importance, especially considering the ever-increasing number of patients diagnosed with the aid of [^18^F]FDG, the number of PET centres and the availability of PET/CT or PET/MR scanners at hospitals. The stability of a drug is defined by its ability to maintain its properties during storage and use, as well as the rate at which the changes in these properties take place. The purpose of stability testing is thus to determine how a drug's quality is affected over time and under various conditions such as temperature, relative humidity, light, etc. A drug's shelf life can be defined as the period in which its stability and thus its efficacy and safety is found to be sufficient (ICH 2003a). The European Pharmacopoeia (Ph. Eur.) states that a preparation must be in accordance with the monograph throughout the shelf life of the product (Council of Europe 2019). PET radiopharmaceuticals often have a very short shelf life due to the short half-life of the PET radionuclides. Therefore, it may seem unnecessary to examine the stability of a product containing a radionuclide with a defined half-life and often a lifetime of less than a normal working day. However, several stability factors can affect the radiolabeled product during the lifetime, which is why it is important to examine stability.

The main routine production of [^18^F]FDG uses saline with a small percentage of ethanol, phosphate or citrate buffer in the synthesis. A two-centre study investigated the use of phosphate buffer in GE Healthcare’s FASTlab synthesis cassettes module. It found that phosphate buffered reagent kits formed precipitation due to aluminium phosphate and thus did not recommend the use of phosphate buffered reagent kits even though another study found them to give the same radiochemical yield (Huang et al. 2016; Long et al. 2013). The main stability issue is radiation-related radiolysis of the active substance and solvents (Jószai et al. 2019; Buriova et al. 2005). These stability issues highlight the importance of this study.

## Quality measurements and [^18^F]FDG stability studies

Ph. Eur. states that a preparation must be in accordance with the monograph throughout the shelf life of the product. Furthermore, the authorities should assess stability based on experimental stability data (Council of Europe 2019).

The European Medicines Agency (EMA) follows the published guidelines for stability testing and retesting by the General International Council for Harmonization of Technical Requirements for Registration of Pharmaceuticals for Human Use's (ICH) (ICH 2003a; b). The guideline is divided into two sections, which deal with new "Drug substance" and "Drug product" respectively. Each section covers principles of stability testing and includes sub-sections on general considerations from the literature and experience, stress testing (active substance), photostability (product), sampling, primary/secondary packaging, specification, test frequency, storage conditions, stability commitment, evaluation, and guidance/labelling for use. There is also an extended version of this guideline which describes stability testing of existing substances and related finished products, guidelines for stability testing, and stability testing of active substances and related finished products. This extension contains the same sections but applies to products that have already been approved with the same active substance. However, there are exceptions, including radiopharmaceuticals, which must be treated in accordance with the guideline in the main document. Furthermore, EMA have made a guideline for radiopharmaceuticals where stability is mentioned since the general stability guidelines are not fully applicable for ready-for-use radiopharmaceuticals, radionuclide generators and radioactive precursors. The most important aspects EMA mentions and should be taken into special consideration are: The minimum and maximum amount of concentration of radioactivity at the time of manufacture. The stability results should be presented on three batches, considered for the upper limit for a batch size. The specific characteristics for the radiopharmaceuticals decide the specification and test procedure. Testing frequency is based upon the shelf-life. For ready-for-use radiopharmaceuticals the shelf life after the time of manufacture should be established (European Medicines Agency 2009).

In their guidance for GMP for PET drugs, the US Food and Drug Administration (FDA) describes the test regimen that should be established, followed and maintained to determine the stability characteristics of an individual tracer. The methods used must be credible, meaningful and specific. Samples for stability tests must be representative of the batch from which they are taken and must be stored under suitable conditions. Test results must be documented and should be used to define sufficient storage conditions and expiration dates for each individual product produced (U.S. Food and Drug Administration 2011, 2015). The FDA's guideline for stability parameters includes radiochemical identity and purity (including levels of radiochemical impurities), visual control, pH, and chemical purity, as well as possible control of stabilizer and/or preservative effect. It is recommended to use stability tests that can distinguish between impurities and degradation products. Furthermore, stability studies should be performed on the product with its highest radioactive concentration and in the correct volume for the final product in the intended packaging. A minimum of three productions of the finished product must be examined over a period of time corresponding to the applied shelf life (U.S. Food and Drug Administration 2011).

## Materials and methods

### Literature study

The non-systematic literature study was based on Ph. Eur 10.0., EMA guideline Q1A (R2) 3, the book *Basic Science of PET Imaging* (ICH 2003a; Khalil 2017), and the FDA's guidance for GMP and stability for PET drugs (U.S. Food and Drug Administration 2011, 2015), and was supplemented with an online non-systematic literature search performed using PubMed and Google Scholar as databases. The keywords used were FDG + Stability and the search was limited to contain keywords in title. Results were limited to studies published in English between 2009 and November 2021. Articles describing clinical findings were not included. Relevant references from the primary articles in the literature search were assessed and included if they contributed information that was relevant to this study.

### Experimental

Experimental work was carried out at the PET-radiopharmaceutical production in The PET Imaging Centre, Dept. of Nuclear Medicine, University Hospital of Central Norway, Trondheim, Norway and at the PET-radiopharmaceutical production in The PET Imaging Centre, University Hospital of North Norway, Tromsø, Norway.

Both PET-production sites have undergone a full GMP validation recently, due to the build of completely new production facilities and is recognized as compliant by the Norwegian Health Agency, granting production licenses. Specifically for analytical methods, tests were carried out according to Ph.Eur when applies and in accordance with the ICH Q2 R1 (ICH 2003a).

In our two-centre stability study we examined the stability of [^18^F]FDG using the Füchtner`s modified Hamacher synthesis with basic hydrolysis. We studied the citrate buffered cassettes provided by GE Healthcare routinely used at production sites (Hamacher et al. 1986; Füchtner et al. 1996). Stability studies were performed on validated quality control equipment for [^18^F]FDG production.

### Chemicals

[^18^O]H_2_O water (enrichment > 98%, GMP quality) was purchased from Rotem GmbH (Israel). MilliQ (type 1) water was used. All synthesis cassettes, dispensing kits were purchased from GE Healthcare. Standards such as FDG, FDM and Glucopyranose were purchased from ABX Advanced Biochemical Compounds GmbH (Germany). All other chemicals were purchased from VWR (Norway) and grade was analytical or better. All reagents and materials were handled according to GMP.

### [^18^F]FDG synthesis

In short, [^18^F]FDG was prepared by the Füchtner’s modified Hamacher method in an automated synthesis module (GE FASTLab2) in a hot cell. The synthesis in general can be described as follows: [^18^F^-^]fluoride ions was produced by bombardment of [^18^O]H_2_O using a GE PETrace 16.5 MeV cyclotron. Water containing [^18^F^-^]fluoride ions was trapped by an anion-exchange column and eluted by a mixture of aminopolyether (Kryptofix) and potassium carbonate. Furthermore, the mixture was azeotropically dried by the addition of acetonitrile, heating, addition of pressure and flow of nitrogen. The reaction was heated for the nucleophilic substitution of [^18^F^-^]fluoride ions on the mannose triflate. Preliminary purification was performed by passing the mixture thorough a column and washed with water several times. The retained intermediate was then deprotected using basic hydrolysis by sodium hydroxide at ambient temperature. The alkaline solution was then pH-adjusted with hydrochloric acid and sodium citrate. Purification was performed with a reverse phase column (Sep-Pak C18) and an alumina cartridge. The purified product was collected through a 0.22 µm filter in sterile glass vials into a lead/wolfram (Tungsten) container (Füchtner et al. 1996). The synthesis had a combined decay corrected radiochemical yield of 81.4% ± 4.6% from end of bombardment (EOB) to end of synthesis (EOS).

### Stability studies

The two different PET centers applied the same stability parameters for their [^18^F]FDG stability studies. In general, the quality analysis included 5 QC samples of 1 mL. A full QC analysis was performed and included all stability tests at time points 0 and at 12 hours. Additionally, at time point 12 hours, the full patient vial was tested (12 mL), 20 mL is the maximum recommended dose in milliliters (V) as defined by Ph. Eur. Reasons for not evaluating a full patient vial for all test timepoints were related to the ALARA (as low as reasonably achievable) principle for radiation protection. At time points 3, 6 and 9 hours a reduced QC was undertaken, using HPLC (chemical identity), pH, HPLC (FDG in product), residual solvents (ethanol and acetonitrile), sterility, HPLC (radiochemical purity), HPLC ([^18^F]FDM), TLC (other radiochemical impurities and TLC (Radiochemical purity [^18^F]FDG + [^18^F]FDM). AT the time points 3, 6 and 9 hours visual control, MCA for radionuclide identity [^18^F]fluoride, radionuclide half-life t ½, spottest for kryptofix, endotoxins and MCA for radionuclide purity [^18^F]fluoride were excluded as they seemed redundant and unnecessary as evaluated by our quality risk assessment at the two centres. The activity to be tested was 4 GBq/mL EOS for both production sites. We used 160 GBq (Trondheim) and 175–178 GBq (Tromsø) at EOB to have room for higher activities when asked for. In the preceding processes of validation, the PQ, there was performed testing using a bracketing approach of high and low activity in order to obtain a robust product process, satisfying the clinical demand and leave a good margin for QC to work with. In the stability studies we opted for a high EOB activity, to be able to deliver higher activities when asked for. Production details from the PET centers in Trondheim and Tromsø are given in Table 1.

**Table 1:** Production details from the PET-centers in Trondheim and Tromsø

Production details	Batch					
	FDG191211015 (Trondheim)	FDG191212016 (Trondheim)	FDG200114004 (Trondheim)	FDG210824 (Tromsø)	FDG210901 (Tromsø)	FDG211012 (Tromsø)
EOB activity (GBq)	160	160	160	175	176	178
Mean target current	47.42	48.8	46.86	41.37	44.96	50.64
Timepoint EOS	07:29:37	07:28:30	07:28:45	07:24:06	07:25:27	07:23:53
Timepoint bulk measurement	07:47:32	07:38:28	07:35:10	07:30:14	07:31:41	07:29:45
Activity in bulk (GBq)	96.06	103,586	99.47	117.14	120.37	108.49
Volume bulk (mL)	19.33	20,33	22.29	21.79	21.49	21.28
Concentration in bulk at EOS (GBq/mL), pre-dilution	5.56	5,43	4,65	5.59	5.83	5.29
Timepoint measured patient vial	08:13:08	08:08:07	08:25:37	07:54:41	07:58:23	07:52:58
Activity patient vial (GBq)	37.09	40.3	36.52	37.21	36.82	38.38
Volume patient vial (mL)	11.7	12.32	12.46	10,7	10,14	10,09
Concentration patient vial at EOS (GBq/mL)	4.17	4.20	4.20	4.22	4.47	4.57
Concentration patient vial at dispensing (GBq/mL)	3.17	3.27	2.93	3.48	3.63	3.80

Pre-dilution is fixed at 1mL

### [^18^F]FDG quality controls

HPLC analysis was performed on an Agilent 1260 Infinity II BioInert HPLC equipped with a LabLogic LogiCHROM ECD detector and a LabLogic FlowRAM radiodetector, controlled with Laura software V.5. The analysis was performed by 20 µL injections on a Dionex 4 × 250 mm CarboPac PA10 column with CarboPac 4 × 50 mm guard at 25 °C using 0.1 M NaOH isocratic gradient with 1 mL/min flow for 14 min. System suitability test was performed with a solution of FDG and FDM standard in MilliQ water both with 25 µg/mL concentration.

TLC analysis for radiochemical purity was performed on Merck Silica gel 60 F_254_ 50 × 100 mm, developed over 9/10 of the plate with mobile phase 5:95 MilliQ:MeCN. 2 µL QC-sample was added to the plate. For the system suitability test a 2 µL solution of 30 mg/mL1,2,3,4-tetra-O-acetyl-β-D-glucopyranose standard (ABX) and 20 mg/mL Glucose standard (VWR) in MilliQ-water was added in a separate spot. Radiochromatogram was conducted using a LabLogic Dual ScanRAM radio TLC scanner followed by visualisation of the system suitability test using 75 g/L sulphuric acid in MeOH developing and heat.

TLC analysis for impurity B, kryptofix was performed on Merck Silica gel 60 F_254_ 50 × 20 mm pre-treated with Iodoplatinate reagent R1 (European pharmacopoeia) On the TLC plate 2.5 µL of QC sample, MilliQ water, reference B (Ph. Eur.) and QC-sample + reference B (Ph. Eur.) was added in 4 separate spots and compared after drying.

GC-FID analysis was performed using an Agilent 7697A Headspace Sampler with Agilent 7820A Gas Chromatograph System equipped with a J&W HP-INNOWax, 30 m, 0.32 mm, 0.25 µm, GC column. Analysis incorporated a validated short version of the Ph.Eur. method using 50 °C hold time 5 min, ramping 30 °C/min to 120 °C, flow 3 mL/min. Helium gas as a mobile phase with inj.port 140 °C and FID 250 °C. HS settings were preconditioning 80 °C for 15 min, loop 90 °C, transfer line 100 °C (105 °C PET centre in Tromsø). The injection volume was 1000 µL. Split mode, split ratio 10:1 (5:1 PET centre in Tromsø). System suitability was performed running 3 samples containing 50 µL solution of ACN 0.04% and EtOH 0.5% diluted to 5000 µL with MilliQ-water. A 50 µL QC sample was also diluted to 5000µL volume with MilliQ-water before analysis.

Bacterial endotoxins were determined on an Endosafe Nexgen PTS by Charles River Laboratories using FDA approved Endosafe PTS cartridges with 0.05 EU/mL sensitivity. The QC samples were diluted 1:50 (20 µL (QC-sample) in 980 µL LAL reagent water). Twenty-five µL was added to the four wells on the cartridge in the Endosafe Nexgen PTS.

MCA analysis was performed using Mirion Canberrra Osprey MCA, equipped with Genie 2K software and Laura. Two µl in an Eppendorf tube from a dilution of 20 µL QC-sample in 980 µL MilliQ water type 1 was added to the MCA and analysed immediately and after 24 hours.

The half-life was measured on the dose calibrator (Capintec CRC-55t-PET). The QC sample was placed in the dose calibrator and measured.

The pH-measurement for the QC-sample was performed on a Mettler Toledo Seven Excellence pH-meter with a Mettler Toledo Inlab Ultra Micro-ISM pH-electrode first calibrated by the buffer solutions pH 4.01, 7.00 and 9.21.

A total of 5 QC samples with aliquots of 1 mL in separate tungsten containers and 1 full patient vial 12mL in tungsten container was used for each experiment. The experiments were performed three times at both production sites. Acceptance criteria for the different tests are given in Table 3.

## Results

The primary literature search described in the method section resulted in the inclusion of 6 articles. The PubMed search identified 157 articles, 5 of which were relevant for the stability of [^18^F]FDG. Fourteen articles were found using Google Scholar, 4 of which were relevant for the stability of [^18^F]FDG, Table 2. Of these, one article did not appear in the PubMed search. Table 2 summarizes the results of the search of the primary literature. These sources were also assessed and included in the literature study if they contributed important information in relation to the study (Table 2).

**Table 2:** Results from the primary literature study and relevant references

Study	Year	Title	Relevant references	References
Ferreira	2009	Stability study of 2-[^18^F]fluoro-2-deoxy-D-glucose ([^18^F]FDG) stored at room temperature by physicochemical and microbiological assays	Fawdry (2007)	Ferreira et al. (2009)
Hjelstuen	2011	Standardization of fluorine-18 manufacturing processes: New scientific challenges for PET	Jacobson et al. (2009)	Hjelstuen et al. (2011)
Walters	2011	Stability evaluation of [^18^F]FDG at high radioactive Concentrations	Jacobson et al. (2009), Fawdry (2007), Yu (2006)	Walters et al. (2011)
Dantas	2013	Radiolysis of 2-[^18^F]fluoro-2-deoxy-D-glucose ([^18^F]FDG) and the role of ethanol, radioactive concentration and temperature of storage	Jacobson et al. (2009)	Dantas et al. (2013)
Rahmani	2017	Synthesis, quality control and stability studies of 2-[^18^F]Fluoro-2-Deoxy-D-Glucose(^18^F-FDG) at different conditions of temperature by physicochemical and microbiological assays	Hamacher et al. (1986), Hung (2002), Fawdry (2007), Ferreira et al. (2009), Yu (2006)	Rahmani et al. (2017)
Joszai	2019	Recommendations for selection of additives for stabilization of [^18^F]FDG	Hamacher et al. (1986), Dantas et al. (2013), Meyer et al. (1999), Fawdry (2007), Jacobson et al. (2009), Rensch et al. (2012), Kiselev et al. (2006), Mosdzianowski et al. (2002)	Jószai et al. (2019)
Hamacher	1986	Efficient stereospecific synthesis of no-carrier-added 2-[^18^F]-Fluoro-2-Deoxy-D-Glucose using aminopolyether supported nucleophilic substitution		Hamacher et al. (1986)
Meyer	1999	The stability of 2-[^18^F]fluoro-2-deoxy-D-glucose towards epimerisation under alkaline conditions		Meyer et al. (1999)
Hung	2002	Comparison of various requirements of the quality assurance procedures for [^18^F]FDG injection.		Hung (2002)
Kiselev	2002	Stabilization of radiopharmaceuticals labelled with [^18^F]fluoride. US Patent		Kiselev et al. (2006)
Mosdzianowski	2002	Epimerization study on [^18^F]FDG produced by an alkaline hydrolysis on solid support under stringent conditions		Mosdzianowski et al. (2002)
Yu	2006	Review of [^18^F]FDG synthesis and quality control		Yu (2006)
Fawdry	2007	Radiolysis of 2-[^18^F]fluoro-2-deoxy-D-glucose ([^18^F]FDG) and the role of reductant stabilisers		Fawdry (2007)
Jacobson	2009	Radiolysis of 2-[^18^F]fluoro-2-deoxy-D-glucose([^18^F]FDG) and the role of ethanol and radioactive concentration		Jacobson et al. (2009)
Rensch	2012	Microfluidic reactor geometries for radiolysis reduction in radiopharmaceuticals		Rensch et al. (2012)
Long	2013	Comparison of FASTlab [^18^F]FDG production using phosphate and citrate buffer cassettes		Long et al. (2013)

Sixteen articles were reviewed in this literature study. Table 3 summarizes the most important studies, data for activity, ethanol content (as a stabilizer), temperature used for the stability study, reference work/quality requirements and found durability.

**Table 3:** Overview of stability studies with activity investigated, temperature, amount of ethanol, quality references and shelf life.

Study	Activity GBq/mL	Temperature	Ethanol (%)	Quality references	Shelf life
Ferreira et al. (2009)	0.3–0.7	RT (22 °C)	≈ 0.04	USP 31	10 h
Walters et al. (2011)	19.7–22.6 EOS	–	0.2	USP 34	12 h
Dantas et al. (2013)	0.7–4.8	5, 25, 40 °C	0.1-0.4	Ph. Eur. 7ed	16 h
Rahmani et al. (2017)	0.3–0.5	18–23 °C, 35–40 °C	0.012 mg/mL	Ph. Eur. 7ed	10 h
Jószai et al. (2019)	2 and 15	RT	50 mmol/L	Ph. Eur. 9ed	210 min or 15 h
Kiselev et al. (2006)	–	–	min. 0.01%/GBq/mL	–	–
Fawdry (2007)	6.3 and 11.5	RT	0.1	BP 2005	14 h
Jacobson et al. (2009)	up to 14.2	–	0.1	Ph. Eur. 6ed	10 h
Rensch et al. (2012)	4–23	–	–	–	14 h
Long et al. (2013)	2.26–8.8	–	0.2 ± 0.07	USP 34	–

The [^18^F]FDG produced at the two different PET centres was tested for stability as described earlier at the given time points: 0, 3, 6, 9 and 12 hours. All acceptance criteria were met for the two production sites for the production of [^18^F]FDG at time points 0, 3, 6, 9 and 12 hours. Results from timepoint 12 h EOS from both production sites are gives as examples, see Table 4 showing tests performed, acceptance criteria and results.

**Table 4:** Data from the PET centres in Tromsø and Trondheim and showing stability studies for [^18^F]FDG after 0, 3, 6, 9 and 12 hours EOS on average of three batches

Tests	Acceptance criteria	Results (Trond-heim) O h	Results (Tromsø) O h	Results (Trond-heim) 3 h	Results (Tromsø) 3 h	Results (Trond-heim) 6 h	Results (Tromsø) 6 h	Results (Trond-heim) 9 h	Results (Tromsø) 9 h	Results (Trond-heim) 12 h	Results (Tromsø) 12 h
Characters Visual control	Clear, colourless or slightly yellow solution	Pass	Pass							Pass	Pass
Identification MCA: Radionuclide identity [^18^F]fluoride	511 keV ± 3%^a^^1^	513.4 ± 2.3	511.2 ± 1.7							512.4 ± 0.6	509.3 ± 0.9
Identification Radionuclide, T_1/2_	105–115 minutes	110.0 ± 0.5	111.3 ± 1.5							109.4 ± 0.7	110.3 ± 0.6
Identification HPLC: Chemical identity	Rt FDG SST, the same as Rt for [^18^F]FDG	Pass	Pass	Pass	Pass	Pass	Pass	Pass	Pass	Pass	Pass
Tests pH	4.55–8.45	5.7 ± 0.3	5.0 ± 0.2	5.6 ± 0.3	5.0 ± 0.2	5.5 ± 0.4	4.9 ± 0.2	5.6 ± 0.2	5.0 ± 0.2	5.5 ± 0.3	5.16 ± 0.1
Tests HPLC: FDG in product	< 25 µg/mL^b^	0	0	0	0	0	0	0	0	0	0
Tests Spottest: Kryptofix	< 0.11 mg/mL^b^	Pass	Pass							Pass	Pass
Tests Residual solvents GC: Ethanol	2.5 mg/mL^b^	1.53 ± 0.2 mg/mL	1.42 ± 0.3 mg/mL	1.68 ± 0.4 mg/mL	1.42 ± 0.5 mg/mL	1.63 ± 0.5 mg/mL	1.40 ± 0.3 mg/mL	1.64 ± 0.5 mg/mL	1.53 ± 0.4 mg/mL	1.61 ± 0.5 mg/mL	1.36 ± 0.4 mg/mL
Tests Residual solvents GC: Acetonitrile	0.205 mg/mL^b^	< 0.103 mg/mL	< 0.064 mg/mL	< 0.103 mg/mL	< 0.064 mg/mL	< 0.103 mg/mL	0.070 ± 0.01 mg/mL	< 0.103 mg/mL	0.085 ± 0.04 mg/mL	< 0.103 mg/mL	0.08 ± 0.03 mg/mL
Tests Sterility	0 CFU/mL	Sterile	Sterile	Sterile	Sterile	Sterile	Sterile	Sterile	Sterile	Sterile	Sterile
Tests Endotoxins	< 8.75 EU/mL^b^	< 2.50	< 2.50							< 2.50	< 2.50
Tests MCA Radionuclide purity [^18^F]fluoride	> 99.9%	100%	100%							100%	100%
Tests HPLC: Radiochemical purity, [^18^F]FDG + [^18^F]FDM	> 95 %	99.6 ± 0.004 %	99.6 ± 0.025 %	99.2 ± 0.001 %	99.1 ± 0.180 %	99.0 ± 0.002 %	98.1 ± 0.936 %	99.0 ± 0.003 %	98.7 ± 0.494 %	98.3 ± 0.003 %	98.7 ± 0.7
Tests HPLC: [^18^F]FDM	< 10 %	0%	0%	0%	0%	0%	0.25 ± 0.388	0%	0.053 ± 0.092	0 %	0.08 ± 0.1
Tests TLC: Other radiochemical impurities	< 5%	0.34 ± 0.006%	0.36 ± 0.09%	0.00 ± 0.00%	0.13 ± 0.115%	0.54 ± 0.009%	0.28 ± 0.242%	0.46 ± 0.008%	0.8 ± 0.397%	0.38 ± 0.007%	0.5 ± 0.4
Tests TLC: Radiochemical purity [^18^F]FDG + [^18^F]FDM	> 95%	99.7 ± 0,01%	99.4 ± 0,41%	100 ± 0.0%	99.6 ± 0,39%	99.5 ± 0.01%	99.4 ± 0,49%	99.5 ± 0.01	98.7 ± 0,74%	99.4 ± 0.08%	98.9 ± 0.7
Tests MCA: Radionuclide purity 24h +	> 99.9%	99.981 ± 0.021	100%							99.987 ± 0.004%	100%

^a^PET Centre in Tromsø 511 KeV ± 5%

^b^Based on 20 mL maximum volume

## Discussion

PET radiopharmaceuticals have a short shelf life due to the short half-life of the PET radionuclides. However, due to stability issues and especially radiation-related radiolysis of the active substance and solvent, it is still important to evaluate several stability parameters in the product's lifetime (ICH 2003b; U.S. Food and Drug Administration 2011). Herein we discuss legislation, guidelines, recommendations, limitations, literature, and our own two-centre stability studies.

After reviewing guidelines and recommendations such as the USP, Ph. Eur., and guidelines from the national regulatory authorities in Norway, the FDA and the EMA we found several important aspects of stability to evaluate. The FDA's guidelines suggest specific stability parameters to be checked in such a study of radiopharmaceuticals, which contrasts with the EMA, which describes stability studies more generally for all current/marketed and future drugs (ICH 2003a; b). EMA describes stability studies of finished products:

- Considerations should be made based on the literature and experience with the active substance and its properties. This includes assessments from stability data on the active substance and evidence from clinical formulation studies.
- Testing for photostability
- At least three units from different batches must be included in the stability study of the finished product, and this must be done for each of the strengths of the product to be marketed.
- The stability study must be performed on the product in the final packaging.
- A specification, namely a list of tests with associated requirements, shall be produced, and confirmed by the stability study. For stability studies of longer duration, there is a requirement to describe test frequency.
- Storage conditions must be specified and supported by the stability study. For studies of longer duration, beyond the time of marketing authorization, the manufacturer must commit to conducting long-term studies of stability. The stability study shall result in an evaluation, which, in addition to the test results for quality, includes degradation products and other relevant conditions that may affect the quality, safety and efficacy of the product.
- Additionally, the product must have a storage guide for the packaging in line with national legislation (ICH 2003a). Storage instructions must be based on data from the stability study. The shelf life will then be defined as the maximum time the product can undergo quality control in accordance with the monograph with satisfactory results. Considerations should be given to which other aspects of storage may affect the shelf life of the product, including photostability, temperature, septum integrity, as well as instructions for use and labelling. The European Pharmacopoeia describes that a product must conform to the monograph throughout its life, which means that parts of the stability study can be carried out by repeated quality control of the product at set times after dispensing. The shelf life of the product will be accurate if it complies with the requirements set out in the pharmacopoeia, in addition to the other requirements described in the EMA guidelines (Council of Europe 2019; European Medicines Agency 2009).

For the stability evaluation of [^18^F]FDG, some important precautions must be taken into consideration and may not apply to other sites; any deviation must be evaluated in a quality risk assessment. First, the [^18^F]FDG production followed GMP standards: validated production method, validated cleanroom, validated personnel working aseptically and with validated equipment. It means the environment is controlled; it is a cleanroom, where temperature (room temperature, 15–25°C), microbiological contaminants and moisture are controlled. Additionally, the experimental study was carried out on fully validated equipment according to ICH Q2 R1 (ICH 2003a), ensuring confidence in reproducibility of the methods and quality of the experimental data. At both production sites, experimental data revealed that the stability for [^18^F]FDG is suitable and accepted for all tests at the time points 0, 3, 6, 9 and 12 hours with the given radio-stabilizer ethanol with 4 GBq/mL activity. As expected, we saw less radiochemical purity at high starting activities and low dilution volumes, however as mentioned the stability was found suitable even at high EOB starting activity. The product ([^18^F]FDG) is documented thoroughly in the literature and is thus a well-known drug. Its photostability was not evaluated as it was considered irrelevant for this study since the product is kept in a container (lead/wolfram(tungsten)) protected from light and is only exposed to light when it is transferred from the container to the injection module right before being injected into patients. Likewise, the sterile evaluation of the multidose withdrawal from each vial was not evaluated since, in our case, the injection into patients was performed by the automated Posijet module. However, sterility can be maintained over multiple withdrawals, even over several days, using sterile techniques (Gallardo et al. 2015). There is also a risk for microbial growth can occur at room temperature vs. at lower temperatures at for instance 2-8 degrees Celsius. Also, residual glucose from the synthesis in the final product can provide nutrition for potential microbiota. However, the product is supposed to be sterile at delivery, the product is tested for sterility even though the result is not known at the time of release. The risk-assessment and validation of the production must ensure that the production method and dispensing is indeed an aseptic process. The final dispensing step is done using sterile filtration and aseptic procedures. Ethanol in the concentrations used in the FDG-production has no or very limited conservational effects on microbiota. Dantas et al. (2013) investigated whether sterility was maintained during repeated withdrawal from a multidose vial containing [^18^F]FDG and found that this practice does not pose a risk to the patient as long as it is performed according to aseptic technique with the right sterile equipment. Furthermore, for our stability studies the activity was set to be 4 GBq/mL at EOS. The stability studies were performed within primary packaging, in our case a labelled vial with septum and cap. The acceptance criteria in our specifications are the same for release and for shelf-life acceptance and are given in Table 3. The acceptance criteria are based on ICH and GMP guidelines (ICH 2003a; U.S. Food and Drug Administration 2011). These criteria can be different for release and shelf-life considerations: for example, the amount of degradation can be different after release and after storage (ICH 2003a; b).

When we started this work it became clear that no updated overview of the stability literature for [^18^F]FDG was available. Therefore, the purpose of the non-systematic literature study was to get an overview of and updated information on the stability evaluations for [^18^F]FDG. We also found some contradictions regarding activity and stabilization with stabilizers we wanted to examine. The starting point of the non-systematic literature study was to find relevant information in relation to PET radiopharmaceutical production and [^18^F]FDG. The literature search was limited to publications in English from the past 12 years that included "FDG" and "stability" in the title. Additionally, literature containing [^18^F]FDG prepared by the Hamacher synthesis by nucleophilic substitution was included. These parameters limited the literature study. However, the purpose of the study was to get an overview and updated information. Furthermore, the relevant references from the primary articles provided deeper insight into the stability of [^18^F]FDG and were found to be sufficient for this literature study. All sixteen studies are listed in Table 2.

It is well known that the major stability issue for radiopharmaceuticals is radiolysis which can be either autoradiolysis, self-destruction by its own radiation, and/or attack by free radicals formed by the radiation on environmental species. The radiolysis, or more specifically autoradiolysis, of [^18^F]FDG mainly results in free [^18^F^-^]fluoride ions (Jószai et al. 2019). The radiolysis of [^18^F]FDG also generates free radicals because of the reaction of ionizing radiation with water (Buriova et al. 2005). Additionally, Buriova et al. reported two other impurities after autoradiolysis during the synthesis of [^18^F]FDG. They were identified as 2-[^18^F]fluoroglucuronic acid and 2-[^18^F]fluorogluconic acid and are products of autoradiolysis. However, they counted for less than 1.3% of the total activity (Buriova et al. 2005). There are several strategies to reduce radiolysis: one common and well documented strategy is to stabilize with radio-stabilizers. The most well-known and frequently used radio-stabilizer is ethanol. Dantas et al. (2013) found that ethanol content of 0.1–0.4% was sufficiently stable hours in terms of radiochemical purity for up to 16. However, no correlation was found between ethanol concentration and radiochemical purity. This contrasts with Jacobson et al. (2009). Similar to Fawdry (2007) and Jacobson et al. (2009), Dantas et al. (2013) showed that the degradation of [^18^F]FDG to free [^18^F^-^]fluoride ions increases until approx. 4 h EOS, after which the increase due to radiolysis is offset by the decay of [^18^F]fluoride and stabilizes at approx. 2%. Walters et al. (2011) found that 0.2% ethanol was needed to keep batches with activity of 19.7–22.6 GBq/mL stable for up to 12 hours after EOS. Moreover, batches of 0.1% and 0.0% ethanol failed radiochemical purity for such high activity (19.7–22.6 GBq/mL) 5 hours and 1 hour, respectively, after EOS. Walters et al. tested their batches according to the USP, which allows a radiochemical purity og > 90%. On average 6% impurities were found at 12 hours post EOS. In our own two-centre stability studies, the ethanol content was set to contain a minimum of 0.1% after 12 hours with the activity of 4 GBq/mL. In a patent registered in the USA, Kiselev et al. (2006) describe that the concentration of ethanol should be 0.01%/GBq/mL. Mosdzianowski et al. (2002) found that ethanol in 0.1% concentration did not have a significant effect on the stability of [^18^F]FDG batches with an activity between 6.3 and 11.5 GBq/mL. In contrast, Jacobson et al. (2009) found that 0.1% ethanol can stabilize batches with activity up to 14.2 GBq/mL for up to 10 h. In another study, Jószai et al. (2019) examined the effect of various stabilizers and found that hydroxyl (OH·) radicals play a crucial role in the radiolysis of [^18^F]FDG and that selective OH· scavengers, such as salicylate, glucose, cysteine and pantothenic acid are good stabilizers. The concentration should be at least 50 mmol/L. Jószai suggests glucose is ideal for stabilizing [^18^F]FDG and found > 98% radiochemical purity after 15 hours. Interestingly, Long et al. (2013), investigated the difference between GE Healthcare FASTLab reagent kits with phosphate and citrate buffer in the synthesis. A higher ethanol content was found in the cassettes with the citrate buffer, and it was concluded that this is better in terms of the stability of the product.

Dantas et al. (2013) studied the stability of [^18^F]FDG at three different temperatures, 5, 25 and 40° C, every two hours for 16 hours. They found that the temperature had no effect on the degradation. Additionally, Ferreira et al. (2009) showed that [^18^F]FDG batches with an activity of 0.3–0.7 GBq/mL are stable for up to 10 hours at room temperature under experimental conditions. The quality requirements that were used as a basis are USP 317. Rahmani et al. (2017) describes that a [^18^F]FDG batch produced with cassettes from ABX at TracerLab and analysed according to Ph. Eur. 7 is stable for up to 10 hours, at room temperature and elevated temperature (35–40 °C).

The dilution factor for the product as a stabilizer must also be considered. Jiménez Romero et al. (2006) recommend physiological saline dilution of [^18^F]FDG preparations. They found a significant difference in the amount of [^18^F] fluoride in undiluted versus diluted product in studies in the period between30 min and 5 h. Hjelstuen et al. (2011) recommends keeping the radioactivity level as low as possible and performing the dilution immediately after EOS; the dilution has limitations due to the maximum volume to be injected, and additionally a radio-stabilizer can be considered.

There have been constant developments in the synthesis of [^18^F]FDG which have improved synthesis, especially with regard to yield and time. Interestingly, Mosdzianowski et al. (2002) investigated whether pH and temperature, as well as the time for the hydrolysis of Acetyl-FDG, have an effect on the amount of [^18^F]FDG formed in the synthesis. They found up to approx. 7 % [^18^F]FDM at extended hydrolysis time (15 min), 2N NaOH and 60 °C, which is still within the requirements of Ph. Eur. Furthermore, Meyer et al. (1999) found that using 0.33 M NaOH, 40 °C reaction temperature and up to 5 min reaction time reduced the epimerization (conversion) of [^18^F]FDG to [^18^F]FDM to 0.5%. In addition, Rensch et al. (2012) investigated the use of microcapillaries to store [^18^F]FDG without high activity stabilization to avoid autoradiolysis and found up to > 80% radiochemical purity for up to 14 hours after EOS for activities from 4 to 23 GBq/mL.

The experimental study at both production sites revealed that the stability for [^18^F]FDG is suitable and accepted for all tests at the time points 0, 3, 6, 9 and 12 hours with the given radio-stabilizer ethanol with 4 GBq/mL activity. In accordance with the quality risk evaluations and also based on the ALARA full QC tests were performed at o and 12 hours as listed in Table 5. Reduced QC-tests were performed at time points 3, 6 and 9 hours, as listed in Table 5. Another stability parameter that was excluded was the temperature since the product is kept stable at room temperature at hospital settings. Therefore, no temperature variations were included. Dantas et al. (2013) and Rahmani et al. (2017) tested different temperatures and found the [^18^F]FDG stable for 5–40 °C, as mentioned previously.

**Table 5:** List of QC stability tests performed at the two production sites.

	0 h	3 h	6 h	9 h	12 h
Characters	Visual control				Visual control
Identification	MCA: Radionuclide identity [^18^F]fluoride				MCA: Radionuclide identity [^18^F]fluoride
Identification	Radionuclide identity, T_1/2_				Radionuclide identity, T_1/2_
Identification	HPLC: Chemical identity	HPLC: Chemical identity	HPLC: Chemical identity	HPLC: Chemical identity	HPLC: Chemical identity
Tests	pH	pH	pH	pH	pH
Tests	HPLC: FDG in product	HPLC: FDG in product	HPLC: FDG in product	HPLC: FDG in product	HPLC: FDG in product
Tests	Spottest: Kryptofix				Spottest: Kryptofix
Tests	GC: ethanol	GC: ethanol	GC: ethanol	GC: ethanol	GC: ethanol
Tests	GC: acetonitrile	GC: acetonitrile	GC: acetonitrile	GC: acetonitrile	GC: acetonitrile
Tests	Sterility	Sterility	Sterility	Sterility	Sterility
Tests	Endotoxins				Endotoxins
Tests	MCA: Radionuclide purity [^18^F]fluoride				MCA: Radionuclide purity [^18^F]fluoride
Tests	TLC: Radiochemical purity [^18^F]FDG + [^18^F]FDM	TLC: Radiochemical purity [^18^F]FDG + [^18^F]FDM	TLC: Radiochemical purity [^18^F]FDG + [^18^F]FDM	TLC: Radiochemical purity [^18^F]FDG + [^18^F]FDM	TLC: Radiochemical purity [^18^F]FDG + [^18^F]FDM
Tests	HPLC: [^18^F]FDM	HPLC: [^18^F]FDM	HPLC: [^18^F]FDM	HPLC: [^18^F]FDM	HPLC: [^18^F]FDM
Tests	TLC: Other radiochemical impurities	TLC: Other radiochemical impurities	TLC: Other radiochemical impurities	TLC: Other radiochemical impurities	TLC: Other radiochemical impurities
Tests	TLC: Radiochemical purity [^18^F]FDG + [^18^F]FDM	TLC: Radiochemical purity [^18^F]FDG + [^18^F]FDM	TLC: Radiochemical purity [^18^F]FDG + [^18^F]FDM	TLC: Radiochemical purity [^18^F]FDG + [^18^F]FDM	TLC: Radiochemical purity [^18^F]FDG + [^18^F]FDM
Tests	MCA: Radionuclide purity 24h +				MCA: Radionuclide purity 24h +

For future [^18^F]FDG implementation, we present some recommendations based on the non-systematic literature study and the two experimental stability studies.

### Highlights and recommendations from our studies

- The shelf life depends on the starting amount of radioactivity, the radioactivity concentration, the content of stabilizers and the storage conditions.
- A thorough risk evaluation should be performed based upon the ICH guidelines including general considerations literature, general considerations experiences, stress testing, photostability, sampling, primary/secondary packaging, specification and evaluation and guidance/label for use.
- The stability evaluations and continuous evaluations or monitoring should have a defined plan
- Activity and the desired shelf-life must be defined by the production site.
- Ethanol is described as an important stabilizer in several studies and similarly investigated in different amounts with varying amounts of activity.
- With higher activities than 4 GBq/mL special care should be taken in monitoring the radiolysis and the side products that may occur.
- Ethanol content should be at least 0.1% up to 4 GBq/mL (> 0.2% for activities up to 22.6 GBq/mL)
- Since GC Residual Solvents are exempt from a preliminary release of an [^18^F]FDG batch, it may agitate for leaving that test out of a stability study. However, because of the stabilizing effect of ethanol and the 0.1% recommendation GC analysis should be conducted throughout the studies, as ethanol will degrade to acetaldehyde and the stabilizing effect will be reduced accordingly.
- Recommended stability tests for the stability evaluations are according to Ph. Eur. and are listed in table 5.

## Conclusions

The formulation and production of [^18^F]FDG today, including choice of basic hydrolysis, pH, time, and temperatures etc., are based on studies and discoveries that are partly summarized in this retrospective non-systematic literature study. The literature study thus confirms that the method used for the radiopharmaceutical production of [^18^F]FDG remains relevant today in light of new knowledge and technology. We have presented available stability data from several studies and even performed our own two independent stability studies which found the [^18^F]FDG stable for 12 hours at room temperature up to 4 GBq/mL using ethanol as a radio-stabilizer.

## Data Availability

The datasets used and/or analysed during the current study are available from the corresponding author on reasonable request.

## Abbreviations

18F: [18F]Fluoride
BP: British pharmacopeia
CT: Computed tomography
CMC: Chemistry, manufacturing and controls
EMA: European Medicines Agency
EOB: End of bombardment
EOS: End of synthesis
FDA: Food and Drug Administration
FDG: Fluorodeoxyglucose
FDM: Fluorodeoxymannose
GC: Gas Chromatography
GMP: Good manufacturing practice
HPLC: High performance liquid chromatography
ICH: International Council for Harmonization of Technical Requirements for Registration of Pharmaceuticals for Human use’s
MCA: Multi channel-analyzer
MR: Magnetic resonance
PET: Positron emission tomography
Ph. Eur.: European pharmacopeia
QC: Quality control
TLC: Thin layer chromatography
USP: United States pharmacopeia
